# Development of a tailored strategy to improve postpartum hemorrhage guideline adherence

**DOI:** 10.1186/s12884-018-1676-6

**Published:** 2018-02-08

**Authors:** Suzan M. de Visser, Mallory D. Woiski, Richard P. Grol, Frank P. H. A. Vandenbussche, Marlies E. J. L. Hulscher, Hubertina C. J. Scheepers, Rosella P. M. G. Hermens

**Affiliations:** 1 Department of Obstetrics and Gynecology, Radboud Institute for Health Science, Radboud University Medical Center, Geert Grootplein 10, P.O. Box 9101, 6500 HB Nijmegen, the Netherlands; 2 Department of IQ Healthcare, Radboud Institute for Health Science, Radboud University Medical Center, Nijmegen, Netherlands; 3 Department of Obstetrics and Gynecology, GROW School for Oncology and Developmental Biology, Maastricht University Medical Center, Maastricht, The Netherlands

**Keywords:** Implementation strategy, Postpartum hemorrhage, Substandard care, Tailor-made

## Abstract

**Background:**

Despite the introduction of evidence based guidelines and practical courses, the incidence of postpartum hemorrhage shows an increasing trend in developed countries. Substandard care is often found, which implies an inadequate implementation in high resource countries. We aimed to reduce the gap between evidence-based guidelines and clinical application, by developing a strategy, tailored to current barriers for implementation.

**Methods:**

The development of the implementation strategy consisted of three phases, supervised by a multidisciplinary expert panel. In the first phase a framework of the strategy was created, based on barriers to optimal adherence identified among professionals and patients together with evidence on effectiveness of strategies found in literature. In the second phase, the tools within the framework were developed, leading to a first draft. In the third phase the strategy was evaluated among professionals and patients. The professionals were asked to give written feedback on tool contents, clinical usability and inconsistencies with current evidence care. Patients evaluated the tools on content and usability. Based on the feedback of both professionals and patients the tools were adjusted.

**Results:**

We developed a tailored strategy to improve guideline adherence, covering the trajectory of the third trimester of pregnancy till the end of the delivery. The strategy, directed at professionals, comprehending three stop moments includes a risk assessment checklist, care bundle and time-out procedure. As patient empowerment tools, a patient passport and a website with patient information was developed. The evaluation among the expert panel showed all professionals to be satisfied with the content and usability and no discrepancies or inconsistencies with current evidence was found. Patients’ evaluation revealed that the information they received through the tools was incomplete. The tools were adjusted accordingly to the missing information.

**Conclusion:**

A usable, tailored strategy to implement PPH guidelines and practical courses was developed. The next step is the evaluation of the strategy in a feasibility trial.

**Trial registration:**

Clinical trial registration: The Fluxim study, registration number: NCT00928863.

## Background

Worldwide postpartum hemorrhage (PPH) is the main cause of severe maternal morbidity (SMM). A recent study in the United States estimated PPH to be responsible for almost half of the cases of SMM (47,6%) [1]. Globally the incidence of PPH is estimated around 10,5% and in high resource countries an increasing trend in PPH incidence has been seen [2]. For example, in The Netherlands the incidence increased from 3% in 2003 to 8% in 2011 in second line care [3].

A review on PPH guideline adherence found that 38% of the women with ≥1500 ml blood loss received substandard care [4]. Substandard health care is often suggested as a possible cause for inadequate reduction of morbidity [5–7]. It seems that evidence-based guidelines are not optimally adhered to, leading to substandard care and a gap between evidence-based medicine and clinical application [8].

Guideline dissemination without a tailored implementation strategy to improve spread among professionals and adherence to guidelines is often ineffective [9]. A review evaluating implementation strategies within the field of obstetrics concludes that a prospective identification of efficient strategies and barriers to change is necessary to improve clinical practice guideline implementation [10]. The strategy choice needs to be tailored to the setting for best possible results, consisting of the right tools to increase guideline adherence. In this paper we describe the development of an implementation strategy for a high resource obstetric setting to improve guideline adherence regarding postpartum hemorrhage.

## Methods

### Setting

The current study is part of the FLUXIM trial [11]. In this trial we developed quality indicators on PPH care (a); studied the adherence of these indicators in actual care (b), and analyzed barriers and facilitators for optimal care among both professionals, women and their partners (c). In the last part of the Fluxim trial the outcomes of these data were aggregated and formed the basis of the development of a strategy to improve guideline adherence.

### Development strategy

**Fig. 1:**
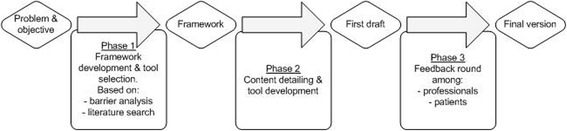
The strategy development process. The three phases for the development of the implementation strategy to improve PPH guideline adherence. The first phase consisted of the analyses of barriers for guideline implementation and the search of international literature of strategy effectiveness, leading to the creating of the strategy framework. The second phase was the content detailing of the created framework and the development of the individual tools. After the first draft was made, a feedback round among professionals and patients was held to assess the content and usability of the tools

The development of the implementation strategy consisted of three phases (Fig. 1).

#### Phase one: The selection of the tools for the implementation strategy

In the first phase the selection of the tools to be included in the implementation strategy was performed by a multidisciplinary expert panel of eight obstetricians, two anesthesiologists and two opinion leaders on quality of care research through an iterative process.

The barriers and facilitators from the professional level chosen for the implementation strategy were those mentioned by at least three out of the four focus groups, and feasible to incorporate in our strategy. These barriers were discussed among the authors to determine which barriers were most likely to supply the greatest gain for improvement and were feasible to include. The same selection criteria were applied to the facilitators. On the patient level, through consensus among the authors, barriers and facilitators were identified as eligible for the strategy.

International literature was searched for evidence on effectiveness of strategies to serve as a base for the selection of the tools to address the barriers and to incorporate the identified facilitators (i.e. the potential tools). The search covered three areas: tools and strategies within the obstetric health care, effective tools outside the field of obstetrics and patient oriented tools. Articles were searched on Medline and experts in the field of implementation science were consulted for recent literature. The search was limited to only article in English, limited to research performed in high resource countries, and there was no date restriction. The search results were presented to the expert panel combined with the barriers and facilitators that were considered most important and the low adherence scores of the actual care study as described above in the setting section [12, 13].

#### Phase two: The development of the tools and their content

In the second phase the selected potential tools were developed. The tool content was derived from international guidelines [14–20], ATLS-based courses (Advanced Trauma Life Support, e.g. the Managing Obstetric Emergencies and Trauma course) and international literature. They give recommendations based on the stage of delivery of the patient, and on the progression of the PPH. There are preventive measures, measures when the blood loss reaches 500 cm^3^ and when there is ongoing blood loss above the liter or 2 l. These phases are also to be found in the division of the quality indicators. The tool set up and content follow this set up of PPH care, and places actions in relation to the stage of amount of blood loss of the patient.

According to both the Dutch and the international guidelines, identification of high-risk patients forms the basis of PPH care. However, most guidelines did not clearly define all risk factors for PPH and there was discrepancy between different guidelines. Therefore an additional search was performed using the Dutch PPH guideline, 6 international guidelines [15–20] and international literature. We searched for additional risk factors and odds ratios (OR’s), and only OR’s with confidence intervals available were considered. Medline was searched using the search terms ‘PPH’ and ‘risk factors’ and synonyms, followed by a snowballing search of the articles and reference lists of the guidelines if available. Furthermore, risk factors found in a multivariate analysis of the Netherlands Perinatal Registration (Dutch Perinatal Registration, DPR) by the LEMMoN study were considered as well (unpublished data, personal correspondence: J. Zwart, Severe Maternal Morbidity in the Netherlands. The LEMMoN study. 2009). Ultimately, all risk factors listed in the Dutch guideline were selected to be included in the tools, as well as all risk factors that were mentioned in at least two of the other six guidelines and found significant in either international literature or in the DPR analysis.

#### Phase 3: Feedback round expert panel and patients

In phase three, the developed tools, was presented in a feedback round among the expert panel. The nine members of the expert panel were asked to evaluate the tools on accuracy of the medical contents, clinical usability and control for inconsistencies with the current best evidence care as provided by the Dutch guideline, and to provide written feedback on these three items.

Patients were recruited to evaluate the patient materials developed for the strategy. Both high-risk patients and patients who experienced a PPH in the previous year were asked for the evaluation. High-risk patients were recruited from the obstetrics clinic in one of the participating hospitals. The patients having experienced a PPH were recruited by placing messages on childbirth forums. The women were asked to evaluate our website by means of a questionnaire. The questionnaire consisted of 37 questions, of which 29 were yes-no questions, evaluating six specific domains, and two general categories with eight open questions for points for improvement. The domains evaluated were the usability, speed of the website, website menu navigation, the completeness and clarity of the information provided, the layout and the risk-identification test available on the website.

## Results

**Fig. 2:**
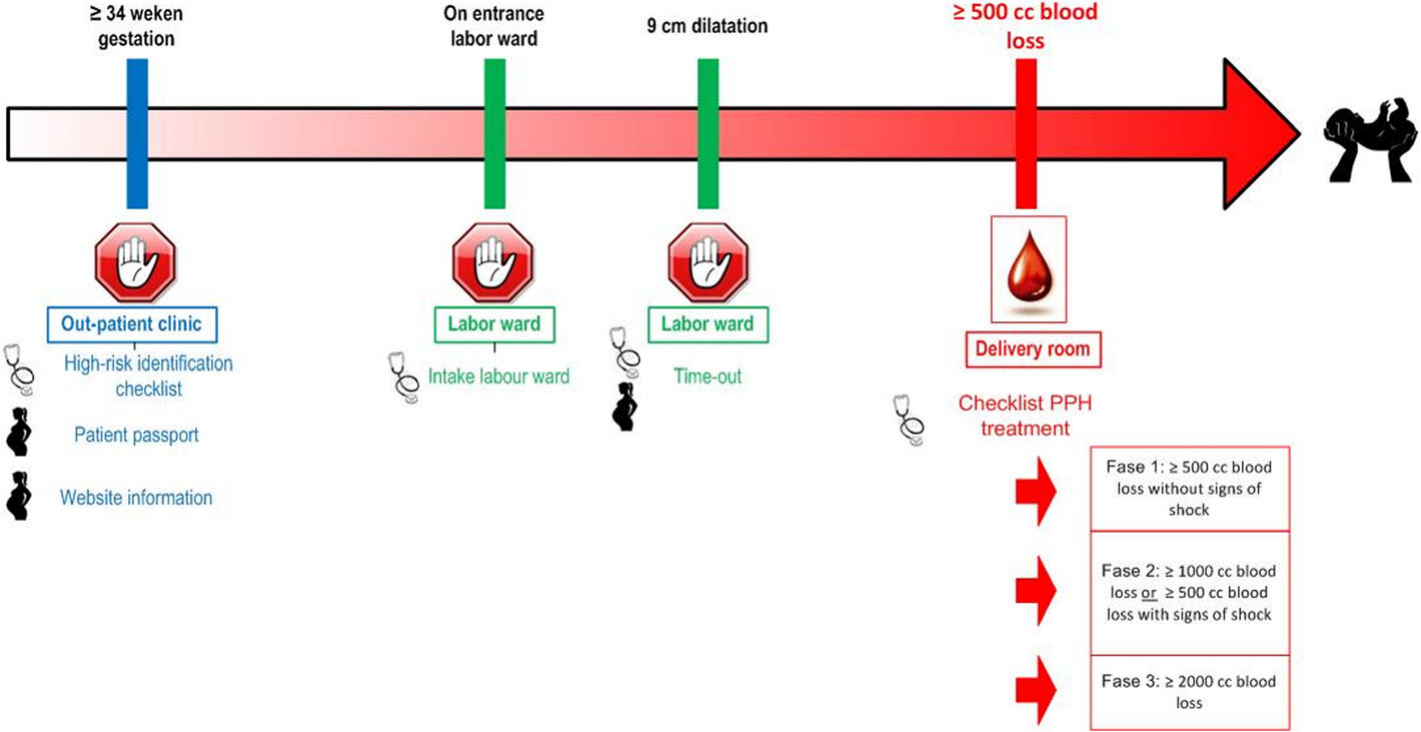
Flowchart of the implementation strategy. Figure gives an overview of the developed, tailored strategy to improve adherence to the evidence-based guideline on PPH care. The strategy covers the trajectory of the third trimester of pregnancy till the end of the third stage of delivery. An extensive description of the content of each of the tools can be found in the results section: Phase two: content detailing and tool development

In the three steps described above we have created a strategy to improve the adherence to evidence based guidelines and the ATLS-based course for prevention and treatment of PPH. The strategy, shown in Fig. 2, consists of three stop moments, a checklist for PPH treatment for the professionals and two patient tools.

### Setting

In short the results of the barriers and facilitators found for optimal PPH care and the current care analysis. The most important barriers experienced by professionals were lack of knowledge, team communication and leadership. They mentioned the use of checklists and flowcharts as factors to improve adherence to the guideline. Patients mentioned lack of information before, during and after the PPH as main barriers and an informative patient website and leaflet as main facilitator for optimal care [12]. Actual care was particularly not in accordance with guidelines with regard to the high risk identification and documentation of policy for PPH on the outpatient clinic and during labor, vital signs monitoring, and the different steps in the management of PPH. Furthermore, acts regarding management of PPH were only partly performed in time [13].

#### Phase one: The selection of the tools for the implementation strategy

The professionals’ barriers and facilitators on which the expert panel reached consensus to include them in the tools, are listed in Table 1, those of the patients are shown in Table 2 [12].

**Table 1:** Main barriers and facilitators addressed by professionals

	Barriers	N^a^
1	Lack of checklist/flowchart about PPH at the delivery rooms	4
2	The guideline is difficult to obtain the at the delivery ward	3
3	Recommendations and definitions in the guideline are unclear	3
4	Professionals overestimate their knowledge regarding identifying the patient-categories at risk for PPH and regarding the treatment of high-risk patients and patients with PPH	4
5	Professionals lack to detect high-risk patients at the outpatient clinic	4
6	Tools: need for practical tools for easier and practical use of the guideline	3
7	Lack of communication in the team responsible for the patient, about the risks, policy’s, seriousness of the situations or actions that need to be taken	4
8	Unclearness in leadership trough lack of knowledge of each other’s skills and ability, because of inexperienced professionals and the frequent change of team composition.	4
9	Disagreement between team members and with personnel of other disciplines about the seriousness of the situation (blood-bank personnel and anesthesiologists)	3
10	Lack of team collaboration, for orders are not followed and team members prefer following their own instincts in treatments that leads to inconsequent policy.	3
11	Presence of hierarchy leads to dread, for team members find it difficult to call in a gynecologist who is at home and speak freely against the supervisor when there is a disagreement about policy	3
	Facilitators	
1	The availability of a checklist/flowchart about PPH at the delivery rooms would improve care	4

^a^ Amount of focus groups that mentioned the barrier or facilitator

**Table 2:** Main barriers and facilitators addressed by patients

	Barriers
1	poor information supply to the patient about PPH
2	poor information supply to the partner and/or family about the medical condition, risks and procedures
3	lack of information material (e.g. folders or website)
4	patient’s perception of delay in transfer to the operation room
	Facilitators
1	information about PPH before the delivery
2	a request for patient information material

When reviewing the literature on strategies for guideline implementation within the field of obstetrics, a systematic review [10] on evidence-based strategies for obstetric guideline implementation provided an overview of effective strategies. Of the tools they reviewed, educational tools showed mixed effects, audit & feedback was generally effective, strategies based on opinion leaders, quality improvement tools and academic detailing were ineffective or showed mixed effects. Reminders showed to be overall effective [10].

Outside the field of obstetrics two types of effective tools were found that seemed applicable in an obstetric setting by the expert panel. The first were checklists, showing to be successful in reducing the complication rate in surgical settings [21–24], and revealing an increase in adherence to safety indicators and guidelines [21, 23]. Checklists can provide an overview in a complicated situation, and reduces room for human error and the number of omitted treatments. Furthermore, checklists can improve documentation of care, facilitate (team- and interdisciplinary) communication and leadership, and minimize information loss during transfer between professionals [23]. Secondly, care bundles, initiated of the Institute for Healthcare Improvement, have in multiple settings shown to increase compliance to quality indicators and reduced complications and mortality [25–27].

Patient empowerment is an important topic in health care and refers to the enhanced ability of patients to actively understand and influence their own health status and health care [28]. According to the WHO, interventions with empowerment characteristics have shown significant impact in improving health and quality of life in chronically ill patients [29]. Web-based interventions seem effective in empowering patients [30] and with a relative young target population a viable option for patient empowerment in PPH care.

Based on the barrier analysis and the literature, the expert panel decided on a multifaceted strategy with separate tools addressing different barriers at different levels, visualized in Fig. 2. The strategy encompassed the complete trajectory starting in the third trimester and ending after the third stage of delivery is finished and the patient is stable. For the professionals, it comprised three stop moments (with a risk assessment checklist, care bundle and time-out procedure), and a PPH treatment checklist. For the patients, a patient-passport was created to provide to high-risk patients and a website for both pregnant and postpartum women to provide preparation information before the delivery and information to process their recent experience.

#### Phase two: The development of the tools and their content

After selection, the individual tools were developed. Standard prototypes of the professional tools were developed based on the latest guidelines and literature.

These prototypes can be adjusted to local protocols (e.g. specific medication choice and dose, telephone numbers of emergency services) before disseminating and implementing them in the different Dutch hospitals. The content of the patient part was written by an obstetrician (MW) with expertise in PPH care. The text was checked by another obstetrician (HS) for accuracy and a layman (RH) for readability and understandability. As the strategy exists of four stop moments, four separate tools were created as can be seen in Fig. 2.

The first stop moment is at the outpatient clinic where the physician has to fill in a risk assessment checklist. The checklist could be filled in from 34 weeks of gestation or beyond. The checklist listed all identified risk factors for a PPH, thus enabling the user to identify high risk patients. It also listed the policy for high risk patients as a reminder for the user.

As described in the methods section a risk assessment checklist was developed by the Fluxim study as there is currently no such list available. To create the risk assessment checklist, in total 34 risk factors were selected for inclusion: 25 risk factors from the Dutch national guideline and an additional nine risk factors were found in at least two international guidelines with significant odds ratios. The risk factors could be divided into four categories: general health history, obstetric history, factors related to the current pregnancy and factors apparent during labor and delivery. The checklist is designed to make the professionals aware of PPH risk factors, alert them on the increased risk and appropriate policy and remind them to inform the patient on the increased risk.

The patient tools that were created for the strategy could be used to inform the patient, facilitating the professional and ensuring consistent and comprehensive supply of PPH patient information. The tools consisted, as mentioned above, of a patient passport that allows patients to identify themselves as high-risk patient to professionals they meet later on in the pregnancy. It also provides written information about PPH and possible preventive measures professionals can take during the delivery. The second tool is a website available to all patients who attended the participating hospitals in the Fluxim trial for their antenatal care.

The second stop moment is upon entrance of the labor ward. At this stop moment professionals need to check if a risk assessment had been performed. In case it has not been done, the checklist has to be provided at that moment.

The third stop moment is near the end of the first stage of labor, closely before entering the second stage. During this stop moment the whole team has to be gathered in the room with the patient for a time-out procedure. It encompasses the checking of the patients’ risk status and corresponding policy, and additional risk factors have to be listed (those that may arise during the first stage of delivery and those possibly arising during the second stage of delivery). As the time-out requires the team to come together in the room with the patient, it stimulates team communication and increases knowledge of all professionals working on the labor ward. The timing of the time-up is left upon the labor ward team, as this might differ per patient depending on the speed of dilatation. A care bundle consisting of preventive interventions (those identified as the active management of the third stage in the Dutch PPH guideline) was incorporated in the time-out, aiding in the standardizing of procedures.

As last of the professional tools, a checklist for PPH treatment was created. This checklist can be used by professionals at a blood loss of 500 ml and ongoing. It guides professionals through consecutive treatment options, gives advice on when to control for what factors (e.g. vital signs, coagulation status, etc), gives an indication of time elapsed and shows when to consult other professionals (i.e. an obstetrician or anesthesiologist). Furthermore, the checklist provides areas for the writing down of times of actions undertaken and vital signs; a procedure advised by ATLS-based courses for Obstetric emergencies.

#### Phase 3: Feedback round expert panel and patients

The expert panel was satisfied with the content and usability of the individual tools of the strategy and there were no points reported concerning inaccuracy of the medical contents, clinical unfeasibility or inconsistencies with the PPH guideline.

Sixteen patients evaluated the website. Of these, 6 were patients within their third trimester of the pregnancy and had an increased risk for PPH, 9 patients had experienced PPH in a recent delivery and of one returned questionnaire it was not clear if the correspondent was pregnant or post partum. Suggestions were made by the patients for improvement of the website, of which the most important improvement was the adding a section about recovery after PPH. All comments were taken into consideration for change and those that were feasible were changed. As some suggestions were not feasible they were not included in the adaptation process. An example of a non-feasible recommendation is the adding of percentages of increased risk per risk factor, which is not feasible as there is no consensus within the literature on the OR’s of the risk factors. Information added to the website included information on the recovery after PPH, information for the partner and information about low-lying placentas. Other changes that were made were the adding of images and suggestion on clearer color schemes.

## Discussion

We developed a tailored strategy to improve adherence to the evidence-based guideline on PPH care within secondary and tertiary care hospitals in the Netherlands. The strategy is based on current care, a barrier analysis and literature. A strategy with 3 stop moments was developed starting in the third trimester of the pregnancy and lasting till the end of the third stage of delivery. Tools used during the three stop moments are a checklist for risk assessment, patient empowerment tools and a time-out closely to the start of the second stage of delivery, with a PPH preventive care bundle incorporated in the time-out. Furthermore a checklist for PPH treatment was developed in case the blood loss exceeded 500 ml postpartum.

Safety checklists, such as the surgical safety checklists, have been derived from aviation and other high-risk industries where they have shown to be effective in reduction of adverse events. The Institute of Medicine published in 1999 the renowned report “To err is human” on medical errors, patient safety and the development of safety systems [31]. They made recommendations to reduce the reliance on human memory and to implement systems that standardize and simplify processes. A checklist is such a system that forces a time-out to summarize the situation and to prepare the professionals for what is coming. It facilitates leadership and open communication, and reduces reliance on memory and the number of omitted procedures. Various types of surgical safety checklists have proven that these systems can be translated to the medical field and successfully reduce complications [21, 22, 24]. A delivery is an acute process where we heavily rely on the memory of the professionals, and where the room for error is large. A recent review on obstetric checklist development confirmed the need to standardize work in the maternity and labor ward, and listed PPH as 6th in their top ten areas that have high priority on checklist development [32].

Involving patients in the perinatal care process creates a shared responsibility and creates opportunity for women to take the lead in the creation of their own care plan. In 2010 an advisory committee (“pregnancy and birth”) of the Dutch Ministry of Health has written a report with advice on how to approach pregnancy and childbirth healthcare from a current and reliable perspective [33]. The aim of the report is to improve (perinatal) health, not solely with the women are sick but in general thus preventing sickness, and to reduce health inequalities. The committee states seven cornerstones, two of which are related to patient empowerment (mother and child in the lead and well informed pregnant patients with shared responsibility). To reach this level of involvement of patients listening to patients and their needs is essential. Including patients in the barrier analysis gave us the opportunity to listen to patients carefully, leading to tools that are actually wanted by patients and filling the current information gap in perinatal care.

Currently, there is a discussion, outside the field of obstetrics, about the added effectiveness of multi-faceted strategies over single-faceted strategies. Although earlier reviews claimed that combinations of many different interventions are often effective [34, 35], Grimshaw found that a higher number of intervention components was not related to higher effectiveness [36].. It seems plausible that combined interventions are only more effective than single interventions, if these address different barriers at different levels. This is also the conclusion of Chaillet et al. [10] Their review shows that in the field of obstetrics multi-faceted strategies are more effective, with the prerequisite that each strategy facet is targeted at its own barrier. Furthermore they showed that a prospective identification of the barriers would enhance its effectiveness, a recurrent finding in reviews on strategy effectiveness [10, 34, 35]. We have created such a multi-faceted, tailor-made strategy with each separate tool developed to address specific barriers.

The framework of our strategy to improve the provision of optimal PPH care in high resource settings is based on barriers found among professionals and patients from the Netherlands, optimizing the strategy for the Dutch setting. However, we believe that the barriers are rather universal, and the framework would thus be applicable in similar obstetric setting in other countries. We detailed the contents of the individual tools in accordance with the Dutch national PPH guideline, international guidelines and literature. As the focus of guideline committees per country can differ, and (conflicting) evidence in literature sometimes leaves room for interpretation, guidelines can vary between countries, organizations and in time. Developing a strategy that is flexible to content and thus adjustable to updates or different surroundings allows it to be constant up-to-date and adaptable for other high-resource countries. As the strategy is low in development cost and maintenance, it could be applicable in low-resource countries, though this still needs to be investigated.

The main strength of our strategy is the fact that it is tailor-made to the field of PPH. Professionals in the field suggested the barriers and facilitators, which most likely facilitates the acceptance of the strategy in a clinical setting.

Limitations of any strategy development lie within the scarce amount of knowledge available for strategy selection. It is known that tailor-made strategies perform better, yet there is no explicit model prescribing which strategy or tool is to be expected most effective in a certain setting. Furthermore limitations of our study are the fact that it is created developed based on barriers found in a high income country, thus limiting the generalization towards lower income countries. Also, our literature search on strategy development evidence was a systematic comprehensive search.

The aim of this article is to describe the process of development, however at this point we need more evidence as to rather the strategy will indeed improve adherence to the guidelines, and ultimately decrease the PPH incidence. Therefore the next steps are testing the feasibility and effectiveness of the strategy in the clinical practice. Before setting up a large randomized controlled trial to evaluate the effectiveness of the trial, a feasibility trial has to be conducted. In such a feasibility trial, the strategy has to be evaluated on usability, time consummation and possible points for improvement. Additionally, an indication towards possible effectiveness and costs can be received. This will allow for optimization of the strategy before testing its cost−/effectiveness in a robust study design.

## Conclusion

In conclusion, to our knowledge, the developed tailored strategy is the first worldwide in the acute setting of obstetric care encompassing the whole process from prevention to treatment of PPH. Based on current barriers and facilitators nominated by professionals in the field combined with international literature we have aimed to create a usable, tailored strategy that can aid in the implementation of evidence-based guidelines into daily practice.

## Abbreviations

ATLS: Advanced trauma life support
DPR: Dutch Perinatal Registration
OR: Odds ratio
PPH: Postpartum hemorrhage
SMM: Severe maternal morbidity
WHO: World Health Organisation
